# Biochar amendment alters rare microbial taxa and enhances wheat growth in alkaline farmland: insights into soil microbiome dynamics

**DOI:** 10.3389/fmicb.2025.1563712

**Published:** 2025-05-21

**Authors:** Jian-Qing Qi, Hai-Yan Yuan, Shu-Chen Sun, Eric Fru Zama, Bao-Xian Tao, Jin Liu, Bao-Hua Zhang

**Affiliations:** ^1^School of Geography and Environment, Liaocheng University, Liaocheng, China; ^2^Department of Agricultural and Environmental Engineering, College of Technology, University of Bamenda, Bambili, Cameroon; ^3^Shandong Academy of Innovation and Development, Jinan, China

**Keywords:** biochar, rare taxa, *phoD*-harboring microorganism, protist, fungi, wheat yield

## Abstract

**Introduction:**

Biochar is recognized as a promising soil amendment for maintaining soil fertility and improving soil conditions. Alkaline farmland is widely distributed globally. Soil microbial taxa, including rare, intermediate, and abundant bacteria, fungi, protists, and *phoD*-harboring microbes, play essential roles in carbon, nitrogen, and phosphorus cycling. However, the impacts of biochar on the community composition of these taxa in alkaline farmland are not well understood. Gaining insights into how the soil microbiome responds to biochar application and its association with crop biomass is crucial for sustainable agriculture. In particular, the responses of rare microbial communities, such as rare protists and *phoD*-harboring microbial taxa, to biochar and their relationship with crop biomass remain largely unexplored.

**Methods:**

In this study, topsoil (0–10 cm) samples were collected from a three-year field experiment in a wheat (*Triticum aestivum* cv. Jimai 22)-maize (*Zea mays* cv. Jiyuan 169) rotational cropping system. The experiment included treatments with and without biochar application (CK). Gene abundance of bacterial 16S rRNA and *phoD*, a gene encoding an alkaline phosphatase involved in phosphorus cycling, was quantified using quantitative polymerase chain reaction (qPCR). The compositions and diversities of bacterial, fungal, protistan, and *phoD*-harboring microbial communities were analyzed by Illumina MiSeq sequencing.

**Results:**

Biochar application significantly reduced soil total phosphorus (TP) and ammonium nitrogen (NH_4_^+^-N) contents. It increased soil N:P ratios by 19.63%, 2.80%, 23.36%, and 27.10% in B0.5, B1.0, B1.5, and B2.0 treatments, respectively. Soil dissolved organic carbon (DOC) positively correlated with bacterial 16S rRNA gene abundance, while total nitrogen (TN) linked to the ratio of *phoD* to bacterial 16S rRNA gene abundance and rare protistan taxa. In terms of crop yield, the B1.5 treatment (3.42 t ha^−1^) increased wheat yield by 35% compared to the CK treatment. Mantel test and random forest analyses indicated that rare *phoD*-harboring, protistan, and fungal communities significantly contributed to wheat growth.

**Discussion:**

This study offers valuable insights into the effects of biochar on soil microbiomes, especially the responses of abundant, intermediate, and rare taxa. The changes in soil nutrient contents and the correlations between soil properties and microbial communities suggest that biochar can modify the soil environment and microbial structure. The significant contribution of rare microbial communities to wheat growth emphasizes their importance in maintaining agricultural ecosystem health and ensuring sustainable ecosystem services. These findings can guide the rational application of biochar in alkaline farmland to promote sustainable agriculture.

## 1 Introduction

Soil microorganisms play a crucial role in soil element biogeochemical processes, such as carbon (C) degradation, nitrogen (N) mineralization, and phosphorus (P) solubilization (Jansson and Hofmockel, 2018; Arenberg and Arai, 2021). Currently, the rare biosphere is attracting the attention of researchers. Previous studies indicated that the entire microbial communities, such as bacteria and fungi, mediate soil ecosystem functions (Liu et al., 2021; Rachel et al., 2018). Nevertheless, in recent studies, rare taxa of those communities have proven to be essential in maintaining soil ecosystem functions and microbial diversity (Wang et al., 2023; Wang et al., 2024; Ma et al., 2024). Biochar, as a soil amendment, influences N-related processes such as ammonium absorption and nitrification rate, as well as processes related to phosphorus transformation, mainly through its interactions with microorganisms (primarily bacteria and fungi) (Clough and Condron, 2010; Lehmann et al., 2011; Zhang et al., 2018; Zhao et al., 2020). Despite their significance in nutrient cycling, particularly P solubilization and soil fertility maintenance, the roles of protists and *phoD*-harboring microbes in the biochar-soil-plant continuum remain poorly understood (Xiong et al., 2020).

Biochar is a porous and highly aromatic substance produced anoxically through the pyrolysis of biomass (e.g., crop waste and lignocellulosic biomass) (Dai et al., 2021). Biochar contains abundant functional groups, including carbonyl (C=O) and carboxyl (–COOH) groups, giving it exceptional adsorption capabilities for both organic and inorganic ions (Zama et al., 2018). Despite these properties of biochar, the effects of biochar on specific microbial groups, such as protists and *phoD*-harboring microbes, have been rarely reported (Yin et al., 2023; Rasit, 2022). Studies have found that biochar significantly influences soil microbial community composition, especially in bacteria and fungi (Shamim Gul et al., 2015; Luo et al., 2017a; Xu et al., 2020; Sarauer and Coleman, 2023). Beyond bacteria and fungi, protists are vital in mediating carbon, nitrogen and phosphorus cycling in agroecosystems (Li et al., 2021; Zhang et al., 2022). Research has primarily focused on how biochar affects soil bacteria, fungi, and specific functional microorganisms involved during nitrogen and carbon transformations (Ameloot et al., 2014; Rutigliano et al., 2014), neglecting its effect on protists in biochar-incorporated soils. Although protists are central in soil nutrient cycling, fertility maintenance, and agricultural ecosystem stability (Asiloglu et al., 2021; Ceja-Navarro et al., 2021; Nguyen et al., 2021; Sun et al., 2021) and are also important bioindicators of soil quality (Xiong et al., 2018), they are overlooked components of agricultural ecosystems with biochar. Consequently, understanding how biochar influences soil protists is of paramount importance.

P, an indispensable nutrient for microbes and plants, determines agricultural productivity and function (Bai et al., 2013; Shi et al., 2015). Only inorganic phosphates are accessible to microbes and plants (Garland et al., 2018). However, inorganic phosphate in the soil combines with iron and aluminum oxides and calcium-containing minerals, forming insoluble inorganic phosphate that accounts for 82% of the total P (TP) in soil and is unavailable to microbes and plants (Parent et al., 2014). In alkaline soils, alkaline phosphomonoesterases (ALPs) are crucial in the release of inorganic phosphate for plant and microbe uptake (Liang et al., 2020). Generally, three homologous genes (*phoD*, *phoA*, and *phoX*; produced by microorganisms) encode phosphatases (Fraser et al., 2015). Owing to its widespread distribution in bacteria and across soil types, the *phoD* gene is considered the most significant alkaline phosphatase-encoding gene (Ragot et al., 2015). Wei et al. (2019) demonstrated that rare taxa of *phoD*-harboring microorganisms were important for mediating P mineralization, while Li et al. (2023) found that they contributed greatly to C, N, and P cycling. Currently, the effects of biochar on these communities remain poorly understood. Therefore, understanding how biochar application affects the abundance and community composition of rare, intermediate, and abundant bacterial, fungal, protistan, and *phoD*-harboring microbe taxa is important for ensuring sustainable agricultural ecosystems.

This field-based study aimed to determine how varying biochar doses affect soil physicochemical properties; rare, intermediate, and abundant bacterial, fungal, protistan, and *phoD*-harboring microbial taxa; and wheat yield. The correlations between soil physicochemical properties, microbial communities, and wheat biomass after biochar application were also assessed. Thus, a comprehensive understanding of the soil microbiome response to biochar incorporation in agricultural ecosystems was obtained. We hypothesized that biochar affects wheat biomass primarily by influencing rare taxa of microorganisms. Understanding how biochar application affects the abundance and community composition of rare, intermediate, and abundant bacterial, fungal, protistan, and *phoD*-harboring microbe taxa and their relationships with wheat yield is important in sustainable agricultural ecosystems.

## 2 Materials and methods

### 2.1 Characteristics of experimental site, soil, and biochar

**Figure 1:**
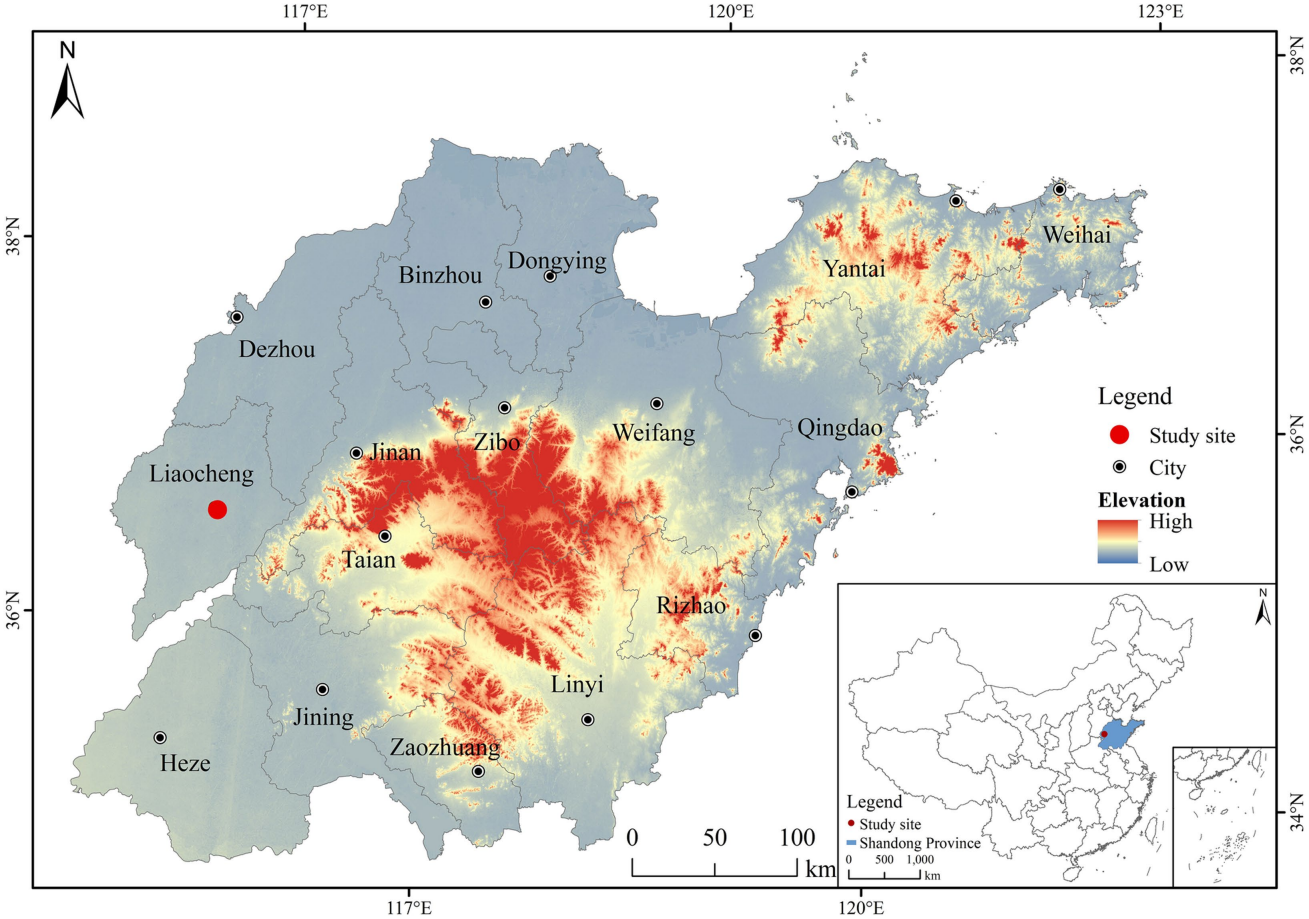
Location of the study area.

The field experiment is situated in western Shandong Province, specifically the wheat (*Triticum aestivum* cv. Jimai 22) and corn (*Zea mays* cv. Jiyuan 169) rotation area at the Soil Experiment Base of Liaocheng University, Shandong Province, China (Figure 1). This area is fluvo-aquic (WRB, 2015) with a soil bulk density, soil field capacity, pH, soil organic matter content, total nitrogen (TN), and soil labile P of 1.50 g cm^−3^, 26.80% (v/v), 8.78, 10.93 g kg^−1^, 0.71 g kg^−1^, and 18.49 mg kg^−1^, respectively. The climate is temperate monsoon climate with continental influences, and the annual average precipitation is 540 mm, concentrated in summer (June to August). The average annual temperature is approximately 13.5°C, while the frost-free period lasts 208 days. The field experiment began in June 2018, and the soil samples were collected in June 2021 throughout this period, wheat and corn were rotated. Wheat was planted from October of each year to May of the following year, while corn was planted from June to September of each year. Field management methods, such as fertilization and pest control, were the same as those of local farmers.

Fresh and uncontaminated corn straw was collected from corn fields after grain harvest. To reduce the moisture content, the straw was first spread out for nature air-drying in a well-ventilated and sunny area. After the moisture content reached approximately 10–15%, the dried straw was cut into 2–5 cm pieces using a sharp-bladed cutter or a forage chopper to ensure uniform heating during pyrolysis. For the pyrolysis of the pretreated corn straw, a muffle furnace was used in the experiment. The cut and dried corn straw pieces were placed in a heat-resistant crucible and loaded into the muffle furnace. The pyrolysis was carried out in an oxygen-limited environment. The heating rate was set at 10°C/min, and the target pyrolysis temperature was 450°C. Once the target temperature was reached, it was maintained for 2 h to allow for complete thermal decomposition of the corn straw’s cellulose, hemicellulose, and lignin into volatile gases, bio-oil, and solid biochar. After pyrolysis, the crucible with the biochar was removed form the muffle furnace and allowed to cool to room temperature in a desiccator. This rapid cooling process was crucial to preserve the biochar’s structure and properties. Subsequently, the cooled biochar was grounded into a fine powder. The grounded biochar was then sieved through a 2 mm mesh sieve to obtain a uniform particle size. The sieved biochar was stored in air-tight containers to prevent re-adsorption of moisture and contaminants prior to its incorporation into the soil. Additionally, the basic properties of the biochar were determined, including pH value of 7.44, TN content of 15.45 g kg^−1^, TC content of 761.20 g kg^−1^, H/C of 0.57%, electrical conductivity of 2,470 μS cm^−1^, available phosphorus content of 1,151.85 mg kg^−1^ and TP content of 4.20 g kg^−1^.

### 2.2 Experimental design

A completely randomized block experiment was performed to obtain five different amounts of biochar. Biochar was applied in accordance with the actual quantity of maize straw produced in a plot. The application rate of biochar was as follows: (1) CK: no biochar addition, (2) B0.5 (1.14 t ha^−1^): 0.5 times the amount of biochar equivalent to the carbonized maize straw produced per plot. (3) B1.0 (2.28 t ha^−1^): 1.0 time the amount of biochar equivalent to the carbonized maize straw produced per plot. (4) B1.5 (3.42 t ha^−1^): 1.5 times the amount of biochar equivalent to the carbonized maize straw produced from per plot. (5) B2.0 (4.56 t ha^−1^): 2.0 times the amount of biochar equivalent to the carbonized maize straw produced per plot. There were three replicates per treatment, resulting in 15 plots in total, with each plot being 8 m^2^.

Biochar was applied in the first year of the experiment and was not added thereafter. Biochar produced from maize straw was evenly spread on the soil surface before sowing winter wheat and then turned into tilled soil. The same amount of fertilizer was applied to each plot, including N (urea, 46% nitrogen, 225 kg ha^−1^), phosphate (P_2_O_5_, 150 kg ha^−1^), and potassium fertilizer (potassium chloride, 51% K_2_O, 150 kg ha^−1^). 50% of the total fertilizer was applied as base fertilizer before summer maize sowing, and the remaining 50% was applied during the jointing stage. The management measures were identical to the local agricultural management measures.

### 2.3 Soil sample collection and analyses

To compare the changes in soil physicochemical properties and microbial indicators of biochar amendment treatments with those of a control treatment, soil samples were collected in June 2021, 3 years after the experiment began. Stem, leaf, tassel, and grain samples, collected in the same year after the wheat had ripened, were oven-dried (70°C) until reaching a constant weight, to determine their dry weights. Fifteen soil core samples were collected at a 0–10 cm depth, completely mixed, and sieved through a 2 mm mesh to remove roots and rocks. The mixed samples were separated into two. The two subsamples were maintained at 4°C and −20°C to assess soil physicochemical properties and for DNA extraction and subsequent analysis, respectively.

The soil pH was determined using a soil and water suspension at a 1:2.5 ratio (w/w). Soil dissolved organic C was analyzed using a TOC analyzer (Vario TOC, Elementar, Germany). Soil TP was measured using the ammonium molybdate method after digestion with perchloric and sulfuric acids. Soil available P was determined using a UV-V spectrophotometer equipped with phosphomolybdate blue. Soil NH_4_^+^-N and NO_3_^−^-N concentrations were analyzed using UV spectrophotometry. Soil TC and TN were measured using an elemental analyzer (Vario MICRO cube, Elementar, Germany).

ALP activity was determined as described by Tabatabai (1994). One gram of fresh soil was incubated for 1 h at 37°C in a pH 11.0 buffer containing *p*-nitrophenol phosphate, after being agitated with 0.5 mL toluene for 15 min. The process was stopped using 0.5 M NaOH. UV spectrophotometry was performed at 420 nm to examine *p*-nitrophenol production. The μg *p*-nitrophenol g^−1^ soil h^−1^ were used to express ALP activity.

### 2.4 Soil DNA extraction and quantification of bacterial 16S rRNA and *phoD* genes

A Fast DNA SPIN Kit for soils (MP Biomedicals, United States) was used to extract genomic DNA from frozen soil samples (0.5 g) according to the manufacturer’s instructions. A NanoDrop ND-2000 spectrophotometer (NanoDrop Co., United States) was used to assess the DNA extracts’ quality and quantity. The abundance of bacterial 16S rRNA and *phoD* genes in the soil samples was measured using quantitative real-time polymerase chain reaction (qPCR) and an iQTM5 Thermocycler (Bio-Rad). The *phoD* gene was quantified using a SYBR Green assay. TaqMan qPCR was used for detecting bacterial 16S rRNA genes. The final step produced a melting curve used to guarantee the amplification specificity of the reaction. The cloned plasmids were diluted 10 times in serial order to create a standard curve. All assay efficiencies exceeded 90%. The conditions and programs for qPCR are shown in Supplementary Table S1.

### 2.5 Illumina sequencing and data processing

Bacteria, fungi, protists, and *phoD*-containing microorganisms were evaluated by Illumina MiSeq sequencing. Three commonly used primer sets were used to study microbial communities, targeting bacterial 16S rRNA and fungal and protistan 18S rRNA genes (Rousk et al., 2010; Stoeck et al., 2010; Fadeev et al., 2021). Details of the primers are shown in Supplementary Table S2. The *phoD* gene was amplified using primers *phoD*-F733 and *phoD*-R1083, following Chen et al. (2019). PCR reactions were conducted in 25 μL mixtures (12.5 μL PremixTaq^TM^, 0.5 μL of each primer, 2 μL of temperate DNA, and 9.5 μL of sterilized ddH_2_O). The PCR conditions for each primer set are listed in Supplementary Table S2. Sequencing experiments were performed at Shanghai Personalbio Technology Co., Ltd. (Shanghai, China). After sequencing, the raw data were uploaded to the Sequence Read Archive database of the National Center for Biotechnology Information (NCBI), and the BioProject IDs were PRJNA970957 and PRJNA1111768.

Protocols by Fadrosh et al. (2014) were used to process raw sequencing read. Barcodes from sequences were extracted during pre-processing, and a TagCleaner was used to clean primer sequences. Quantitative Insights Into Microbial Ecology was used to analyze the filtered reads. Low-quality reads, namely chimeras and erroneous sequences, were removed using quality filtering during ChimeraSlayer and demultiplexing, respectively. Operational taxonomic units (OTUs) were classified with 97% similarity. To eliminate the effect of uneven sequencing depth among samples, sequences in OTU tables were re-sampled to 51,295, 70,452, 58,299, and 40,373 for bacteria, fungi, protists, and *phoD*-harboring microbes, respectively. The OTU taxonomy was identified against the 16S/Silva/v132 database for bacterial sequences, 18S/Silva/v132 for micro-eukaryotic sequences, and NCBI for *phoD*-harboring microbial sequences. Based on research by Jiao and Lu (2020), we defined rare, intermediate, and abundant taxa according to their relative abundances (Jiao and Lu, 2020). Rare taxa with relative abundances of <0.01% the total sequences were analyzed. Conversely, the relative abundance of intermediate taxa fell within 0.01–0.1% (Jiao and Lu, 2020). The most abundant taxa were OTUs with relative abundances exceeding 0.1% of the total sequences (Jiao and Lu, 2020). A total of 769,425 high-quality bacterial reads were obtained and assigned to 24,999 OTUs at a 97% similarity level. In total, 93.37% of OTUs were classified as rare taxa, accounting for 30.43% of the average relative abundance across the entire sample (Supplementary Table S3). In contrast, a small percentage of OTUs (0.43%) were classified as abundant taxa; however, they accounted for 21.82% of the overall sample’s average relative abundance (Supplementary Table S3). The average relative abundance of all the sample’s intermediate taxa, accounting for 6.20% of the total, was 47.75% (Supplementary Table S3). For fungi, 1,056,780 high-quality sequences were obtained, assigned to 3,032 OTUs at a 97% similarity level. Although a large percentage of the OTUs (89.64%) were rare taxa, they accounted for only 3.63% of the average relative abundance across all samples (Supplementary Table S3). Meanwhile, only 2.41% of OTUs were classified as abundant taxa, although accounting for 89.67% of the whole sample’s average relative abundance (Supplementary Table S3). Intermediate taxa comprised 7.95%, accounting for 6.70% of the total sample’s average relative abundance. A total of 874,485 high-quality protist sequences were obtained and assigned to 7,572 OTUs at a 97% similarity level. A total of 12.42% of the average relative abundance of the entire sample was comprised of rare taxa, which accounted for 87.67% of the OTUs (Supplementary Table S3). Only 1.60% of the OTUs were classified as abundant taxa, despite comprising 65.74% of the total sample’s average relative abundance (Supplementary Table S3). The average relative abundance of all the sample’s intermediate taxa, accounting for 10.74% of the total, was 21.84% (Supplementary Table S3). A total of 605,595 high-quality sequences of *phoD*-harboring microbial communities were obtained, assigned to 9,614 OTUs at a 97% similarity level. The majority of the OTUs (88.17%) classified as rare taxa accounted for 15.42% of the average relative abundance across all samples (Supplementary Table S3). Only 1.65% of OTUs were classified as abundant taxa; however, they accounted for 56.25% of the average relative abundances across all samples (Supplementary Table S3). The percentage of intermediate taxa was 10.17%, accounting for 28.33% of the average relative abundance in all the samples (Supplementary Table S3).

### 2.6 Statistical analysis

Unless otherwise stated, all statistical analyses were performed in the R environment,^1^ and most statistics were constructed using “ggplot2,” “vegan,” “ggpubr,” “ape,” “agricolae,” and “randomForest.” We considered *p* < 0.05 to be statistically significant, unless otherwise stated. To evaluate the normality and homogeneity of variance of each edaphic component, we employed the Shapiro–Wilk and Levene tests, respectively. One-way analysis of variance (ANOVA) was used to assess the differences in the physicochemical properties, the abundance of microbial gene copies and the composition of related microbial communities between the biochar treatments.

Non-metric multidimensional scaling analysis (NMDS) based on the Bray–Curtis distance matrices of communities was performed to analyze the abundant, intermediate, and rare bacterial, fungal, protistan, and *phoD*-containing microbe communities’ structures in R using the “vegan” and “ggplot2” packages. And the dissimilarity test of Adonis was utilized to demonstrate the significance of community compositional changes. Mantel’s test was used to evaluate correlations between soil properties factors, wheat yield factors and soil bacterial, fungal, protistan and *phoD*-harboring communities, using the “vegan” package. A machine-learning model (random forest) was used to investigate the robustness of associations between both wheat yield and soil physicochemical properties and microbial taxa represented by NMDS1 using the R package “randomForest.”

## 3 Results

### 3.1 Soil physicochemical properties

Biochar incorporation significantly affected soil physicochemical properties (Table 1). Soil NH_4_^+^-N and NO_3_^−^-N were significantly lower in B2.0 and in both B1.0 and B1.5 than in CK, respectively. Biochar incorporation significantly decreased soil TP in B0.5 and B1.5, whereas differences between biochar treatments and CK for AP were nonsignificant. Soil TN in B1.0, B1.5, and B2.0 exceeded that in CK. Biochar incorporation increased soil N:P by 19.63, 2.80, 23.36, and 27.10% in B0.5, B1.0, B1.5, and B2.0, respectively. No significant differences in pH, DOC, TC, C:N, and ALP activity were observed between the biochar treatments and CK.

**Table 1:** Soil quality attributes among five treatments.

Treatment	pH	NH_4_^+^-N (mg kg^−1^)	NO_3_^−^-N (mg kg^−1^)	TP (mg kg^−1^)	AP (mg kg^−1^)	DOC (mg kg^−1^)	TC (g kg^−1^)	TN (g kg^−1^)	C/N	N/P	C/P	ALP
CK	8.94 ± 0.12a	13.20 ± 0.76a	7.49 ± 0.88ab	964.44 ± 36.32a	8.98 ± 2.50a	31.88 ± 4.56a	17.90 ± 1.10a	1.03 ± 0.05a	16.99 ± 1.75a	1.07 ± 0.07a	17.68 ± 2.77a	84.54 ± 2.29a
B0.5	8.96 ± 0.05a	12.54 ± 0.89a	9.94 ± 1.43b	804.44 ± 45.54b	8.58 ± 1.46a	29.79 ± 1.34a	17.30 ± 0.92a	1.03 ± 0.12a	16.99 ± 1.31a	1.28 ± 0.11a	21.22 ± 2.81a	79.69 ± 0.00a
B1.0	8.81 ± 0.22a	12.71 ± 0.65a	7.18 ± 1.46a	968.25 ± 21.17a	10.94 ± 1.88a	27.48 ± 1.98a	18.30 ± 0.43a	1.07 ± 0.05a	17.26 ± 0.60a	1.10 ± 0.03a	17.85 ± 1.23a	80.77 ± 5.49a
B1.5	8.89 ± 0.04a	11.41 ± 0.83ab	6.30 ± 0.62a	806.99 ± 110.86b	8.04 ± 1.39a	31.22 ± 3.58a	18.20 ± 0.08a	1.05 ± 0.04a	17.14 ± 0.54a	1.32 ± 0.18a	21.70 ± 4.07a	79.15 ± 4.63a
B2.0	8.91 ± 0.12a	10.58 ± 0.59b	7.49 ± 1.01ab	917.46 ± 79.26ab	7.26 ± 0.87a	29.74 ± 3.61a	19.67 ± 2.46a	1.23 ± 0.17a	16.02 ± 1.79a	1.36 ± 0.31a	17.43 ± 0.72a	78.62 ± 2.02a

CK: without biochar incorporation, B0.5: plot amended with 1.14 t ha^−1^ of biochar, B1.0: plot amended with 2.28 t ha^−1^ of biochar, B1.5: plot amended with 3.42 t ha^−1^ of biochar, B2.0: plot amended with 4.56 t ha^−1^ of biochar. Different letters indicate significant differences among different treatments (*p* < 0.05). ALP (mg *p*-nitrophenol kg^−1^ soil h^−1^), potential alkaline phosphatase activity.

### 3.2 The abundance of *phoD* and bacterial 16S rRNA gene and the ratio of *phoD* to bacterial 16S rRNA gene

**Figure 2:**
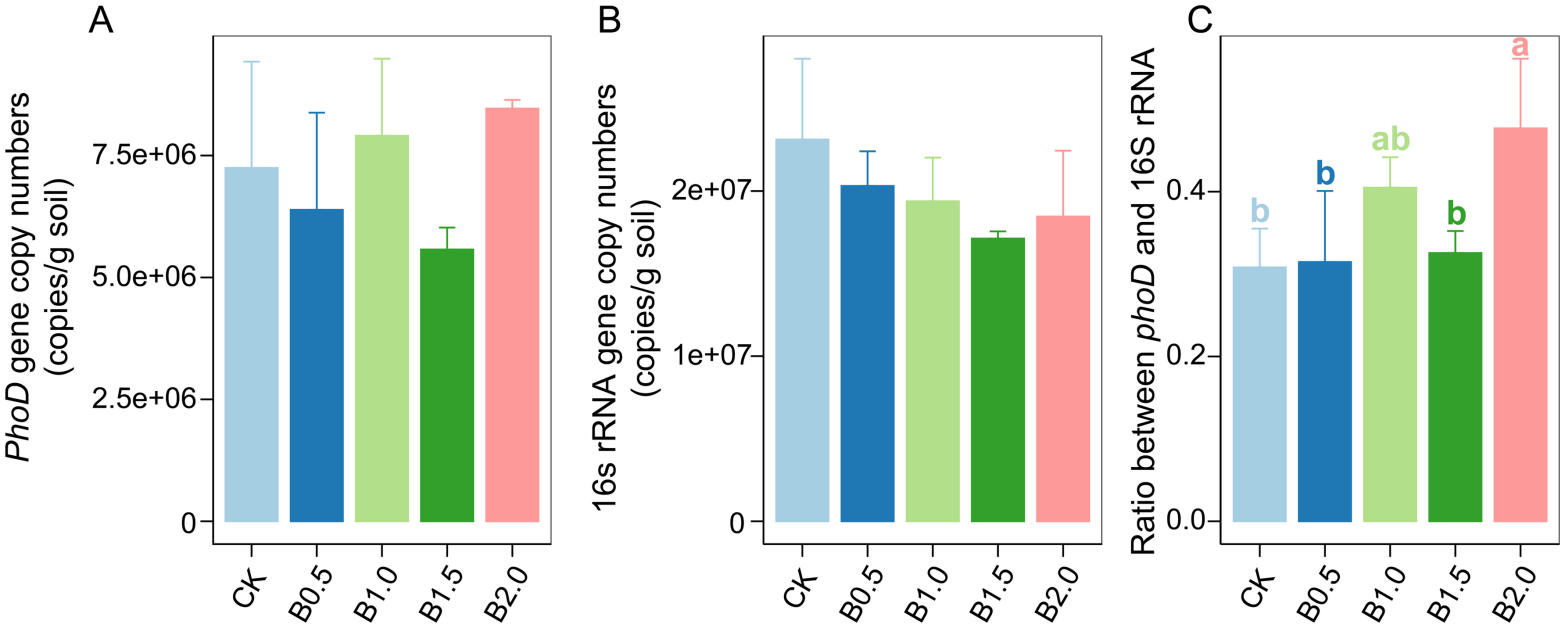
*phoD* **(A)** and bacterial 16S rRNA gene **(B)** and ratio of *phoD* to bacterial 16S rRNA gene **(C)** copy numbers in different treatments. CK: without biochar incorporation, B0.5: plot amended with 1.14 t ha^−1^ of biochar, B1.0: plot amended with 2.28 t ha^−1^ of biochar, B1.5: plot amended with 3.42 t ha^−1^ of biochar, B2.0: plot amended with 4.56 t ha^−1^ of biochar.

The quantitative PCR results showed that *phoD* gene copy numbers were high (2.20 × 10^8^–3.56 × 10^8^ per g of dry soil) in all soil samples (Figure 2A). Biochar incorporation did not significantly alter *phoD* abundance compared with that in CK. The abundance of bacterial 16S rRNA gene copies ranged from 7.31 × 10^8^ to 1.49 × 10^9^ per g of dry soil (Figure 2B). Bacterial abundance did not differ significantly among treatments. However, biochar incorporation significantly increased the ratio of *phoD*-harboring microbes to bacteria (Figure 2C). Compared with CK, higher ratios of *phoD*-harboring microbes to bacterial 16S rRNA genes existed in B0.5, B1.0, and B2.0. Typically, the ratios increased to 31.46, 40.49, and 47.68% for B0.5, B1.0, and B2.0, respectively (Figure 2C). The ratio of *phoD* to bacterial 16S rRNA genes in B1.5 was 32.56%, which was higher than that in CK, although the difference was nonsignificant (Figure 2C).

Mantel’s test was used to investigate associations between soil physicochemical properties and three key metrics: *phoD* gene copies, bacterial 16S rRNA gene copies, and the ratio of *phoD* gene to bacterial 16S rRNA gene copies across the five different treatments. The soil 16S rRNA gene copy number and soil DOC correlated significantly. Furthermore, a positive correlation existed between the ratio of *phoD* gene copy number to bacterial 16S rRNA gene copy number and soil TN across treatments (Supplementary Figure S1).

### 3.3 The structures of bacterial, fungal, protistan, and *phoD*-harboring microbial abundant, intermediate, and rare taxa

**Figure 3:**
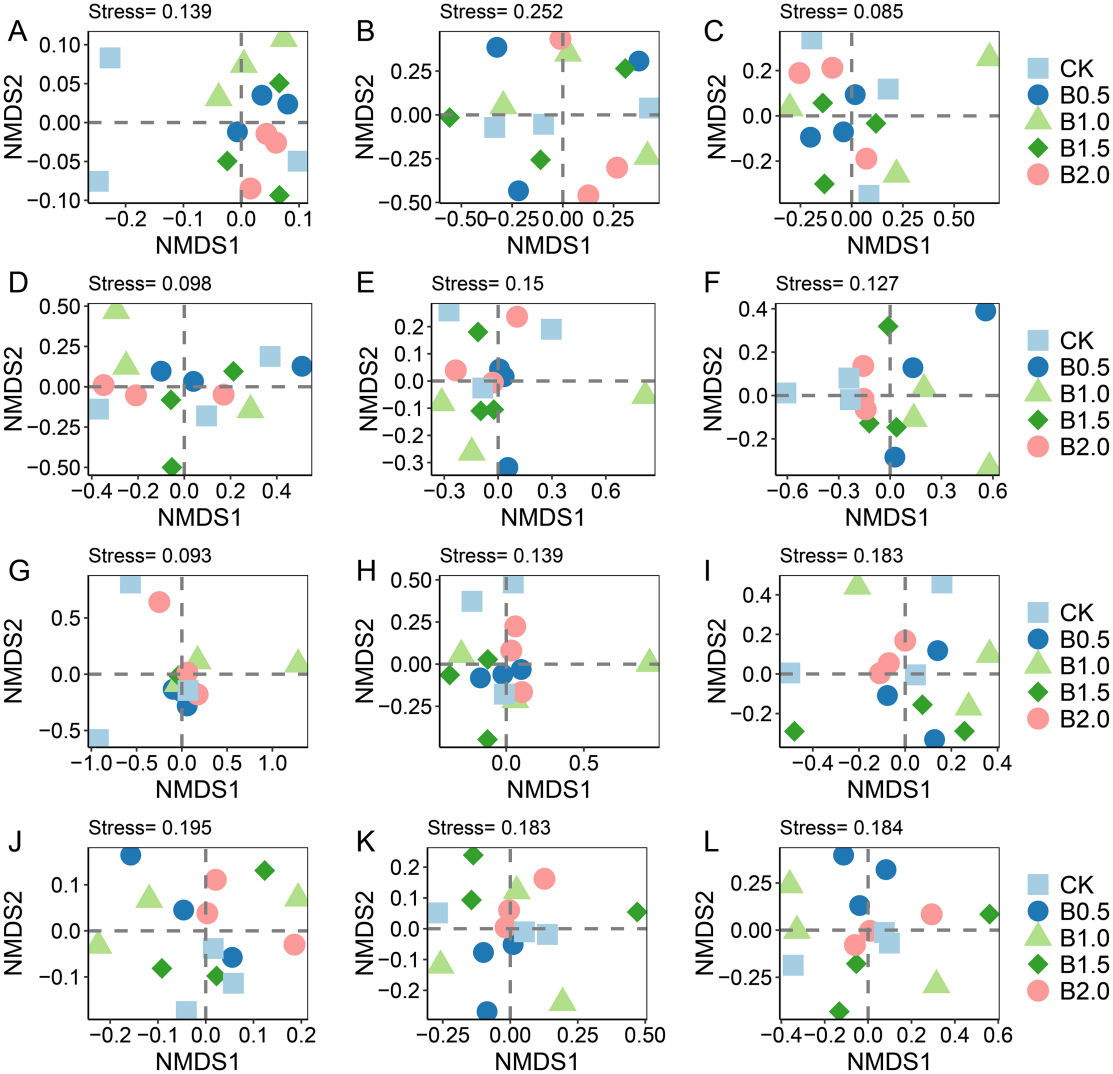
Non-metric multidimensional scaling (NMDS) analysis based on the Bray–Curtis distance matrix for all OTUs was performed on the abundant, intermediate, and rare taxa of bacteria **(A–C)**, fungi **(D–F)**, protist **(G–I)** and *phoD*-harboring microbial communities **(J–L)** in the samples. CK: without biochar incorporation, B1: plot amended with 1.14 t ha^−1^ of biochar, B2: plot amended with 2.28 t ha^−1^ of biochar, B3: plot amended with 3.42 t ha^−1^ of biochar, B4: plot amended with 4.56 t ha^−1^ of biochar.

NMDS-based OTU matrices were used to visualize the differentiation in the abundant, intermediate, and rare bacterial, fungal, protistan, and *phoD*-containing microbe taxa in the five treatments (Figure 3). For bacteria, NMDS clustering of the intermediate and rare taxa showed a large degree of overlap among treatments, implying that the community structures had not changed significantly (Figures 3A,B). Regarding the abundance of bacterial taxa, subsample points of B1.0 were notably distant from those of B0.5, B1.5, and B2.0 (Figure 3C). Species composition analysis shows that Actinobacteria and Proteobacteria were the dominant species in the in the abundant and intermediate bacterial communities (Supplementary Figure S3). And Actinobacteria, Bacteroidetes and Proteobacteria were the dominant species in the in the rare bacterial community (Supplementary Figure S3).

Regarding the relative abundances of rare, intermediate, and abundant bacterial taxa, the relative abundances of intermediate and abundant bacterial taxa were higher and lower in biochar-amended treatments, respectively, than in CK (Table 2). B1.5 showed the highest relative abundance of rare bacterial taxa (Table 2). The links between soil physicochemical properties and bacterial abundant, intermediate, and rare species was investigated using Mantel’s test. No significant correlation existed between the soil physicochemical properties and the abundance of the different bacterial taxa (Supplementary Figure S2A), suggesting that their distribution and abundance may be influenced by factors other than soil physicochemical properties in the studied environment.

**Table 2:** Relative abundance of the abundant, intermediate and rare taxa of bacterial, fungal, protistan, and *phoD*-harboring microorganisms among different treatments.

Taxa	Treatment	Abundant	Intermediate	Rare
Bacteria	CK	0.23 ± 0.01a	0.46 ± 0.01b	0.31 ± 0.02ab
	B0.5	0.22 ± 0.01ab	0.49 ± 0.02a	0.29 ± 0.01b
	B1.0	0.22 ± 0.01ab	0.48 ± 0.01ab	0.30 ± 0.01ab
	B1.5	0.21 ± 0.01b	0.48 ± 0.01ab	0.32 ± 0.02a
	B2.0	0.22 ± 0.01ab	0.48 ± 0.00ab	0.31 ± 0.01ab
Fungi	CK	0.89 ± 0.03a	0.07 ± 0.03a	0.04 ± 0.01ab
	B0.5	0.9 ± 0.02a	0.06 ± 0.01a	0.04 ± 0.01ab
	B1.0	0.91 ± 0.04a	0.06 ± 0.03a	0.03 ± 0.01b
	B1.5	0.89 ± 0.01a	0.07 ± 0.01a	0.04 ± 0.00ab
	B2.0	0.89 ± 0.03a	0.07 ± 0.02a	0.04 ± 0.01a
Protist	CK	0.70 ± 0.12a	0.18 ± 0.08a	0.12 ± 0.04a
	B0.5	0.66 ± 0.03a	0.22 ± 0.03a	0.12 ± 0.02a
	B1.0	0.68 ± 0.11a	0.21 ± 0.06a	0.11 ± 0.05a
	B1.5	0.58 ± 0.04a	0.27 ± 0.05a	0.15 ± 0.01a
	B2.0	0.66 ± 0.11a	0.21 ± 0.07a	0.13 ± 0.03a
*phoD*	CK	0.57 ± 0.02a	0.27 ± 0.01a	0.17 ± 0.03a
	B0.5	0.56 ± 0.02a	0.28 ± 0.01a	0.16 ± 0.01a
	B1.0	0.56 ± 0.03a	0.29 ± 0.03a	0.15 ± 0.02a
	B1.5	0.55 ± 0.02a	0.30 ± 0.04a	0.16 ± 0.02a
	B2.0	0.58 ± 0.03a	0.29 ± 0.00a	0.14 ± 0.03a

CK: without biochar incorporation, B0.5: plot amended with 1.14 t ha^−1^ of biochar, B1.0: plot amended with 2.28 t ha^−1^ of biochar, B1.5: plot amended with 3.42 t ha^−1^ of biochar, B2.0: plot amended with 4.56 t ha^−1^ of biochar. Different letters indicate significant differences among different treatments (*p* < 0.05).

**Figure 4:**
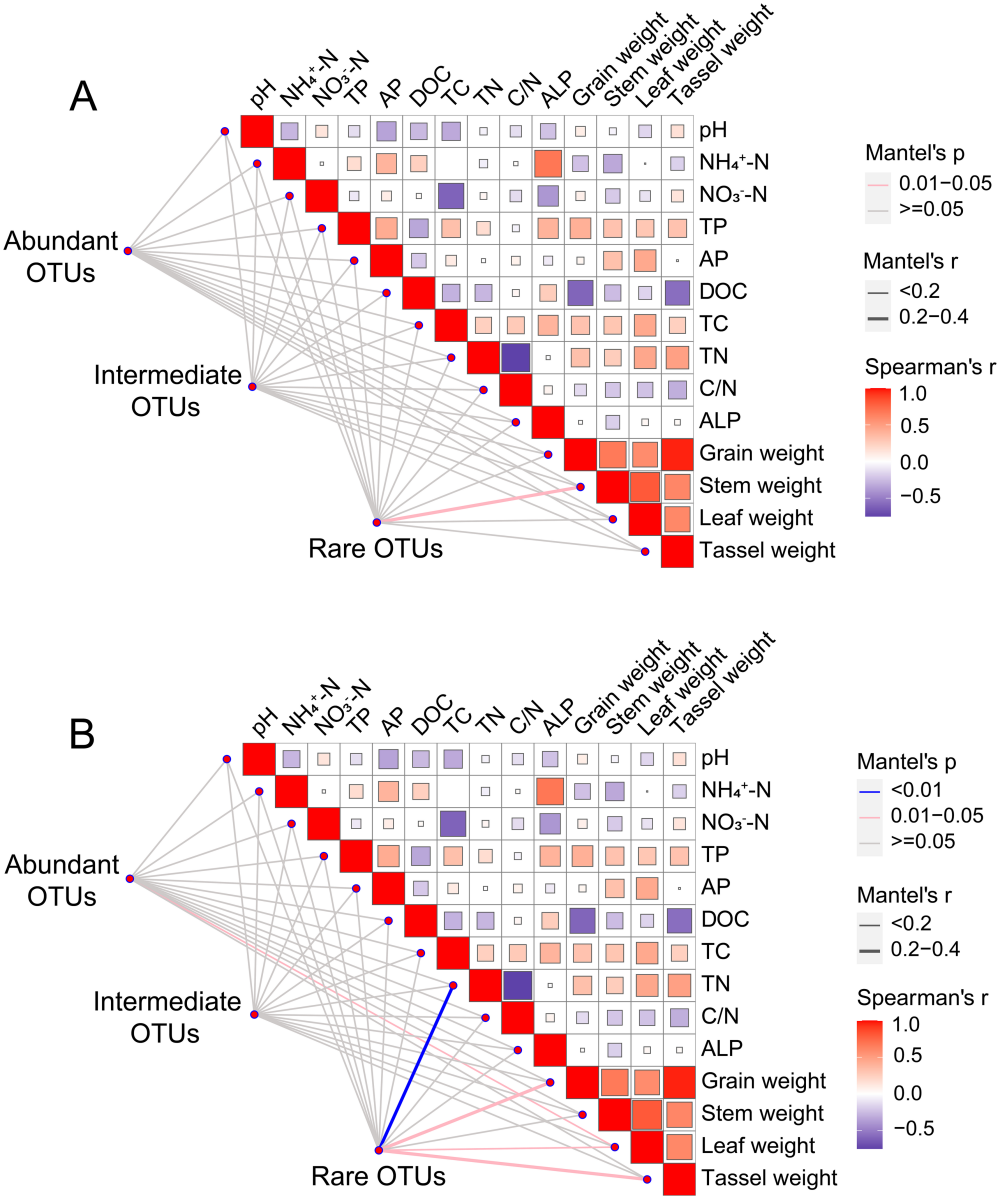
Pairwise comparisons of environmental factors are shown in the upper right corner, with a color gradient denoting Spearman’s correlation coefficient. Abundant (relative abundance >0.1%), intermediate (0.01% <relative abundance <0.1%) and rare OTUs (relative abundance <0.01%) of fungi **(A)** and protist **(B)** are related to each soil attribute and wheat yield by Mantel test. Edge color denotes the statistical significance and edge width corresponds to the Mantel’s *r* statistic for the corresponding distance correlations. TP, total phosphorus; AP, available phosphorus; TN, total nitrogen; TC, total carbon; NO_3_^−^-N, dissolved nitrate nitrogen; NH_4_^+^-N, dissolved ammonium nitrogen; DOC, dissolved organic carbon; ALP, potential alkaline phosphatase activity.

For fungal communities, no significant difference in the abundant and intermediate fungal community taxa existed between treatments (Figures 3D,E). The distribution of sample points for rare fungal taxa was relatively dispersed across different treatments, suggesting that biochar addition had a pronounced effect on the fungal community structure, particularly for rare taxa (Figure 3F). Species composition analysis shows that Ascomycota and Basidiomycota were the dominant species in the in the abundant, intermediate and rare fungal communities (Supplementary Figure S4). Interestingly, we found that the abundance of Protosteliales increased significantly in fungal intermediate taxa. Moreover, the relative abundances of two subcommunities (abundant and intermediate taxa) in the biochar-amended treatments were not significantly different from that of CK, whereas that of rare fungal taxa was significantly lower in B1.0 than in CK (Table 2). Crucial elements influencing the assembly of three fungal subcommunities (abundant, intermediate, and rare) were determined. Mantel’s test was used to assess relationships between soil characteristics and the three fungal subcommunities among the five treatments. No significant correlation existed between the soil physicochemical properties and rare fungal taxa abundance (Figure 4A).

For *phoD*-harboring microbes, Adonis results revealed significant differences between the community structures of intermediate (*p* = 0.092) and rare (*p* = 0.08) *phoD*-harboring microbial taxa among five treatments. NMDS clustering of intermediate and rare *phoD*-harboring microbial taxon communities showed that the sample points of B2.0 did not overlap those of CK and B0.5, implying that the community structures of intermediate and rare *phoD*-harboring microbial taxa changed significantly in B2.0 (Figures 3H,I). Species composition analysis shows that Proteobacteria and Actinobacteria were the dominant species in the in the abundant, intermediate and rare *phoD*-harboring communities (Supplementary Figure S5). And we found that the rare *phoD*-harboring communities were more diverse. Furthermore, we examined the relative abundances of abundant, rare, and intermediate *phoD*-harboring microbial taxa in the five treatments. Although no discernible differences in the relative abundances of abundant, intermediate, or rare *phoD*-harboring microbial taxa occurred between the biochar-amended and CK, the relative abundance of intermediate *phoD*-harboring microbial taxa was higher (Table 2). Mantel’s test was also utilized among the five treatments to investigate the relationship between soil characteristics and *phoD*-harboring microbes, specifically focusing on abundant, rare, and intermediate taxa. AP correlated significantly with *phoD*-harboring intermediate taxa (Supplementary Figure S2B).

NMDS clustering of abundant and intermediate protistan taxon communities showed a large degree of overlap among treatments, implying that the community structure had not changed significantly (Figures 3J,K). The consistently significant distance between subsample points in the rare taxa of the protistan community between CK and B1.5 indicated that biochar incorporation significantly affected the community structure. This suggested that biochar addition caused distinct changes in the rare protist taxa, potentially altering their diversity, abundance, or distribution (Figure 3L). We found alterations in the dominant species of the abundant, intermediate and rare protistan taxon communities (Supplementary Figure S6). Arthropoda was the dominant species in the abundant protistan taxon community (Supplementary Figure S6). Nematoda was the dominant species in the intermediate protistan taxon community (Supplementary Figure S6). And Ascomycota was the dominant species in the rare protistan taxon community (Supplementary Figure S6). The relative abundance of abundant, intermediate, and rare protistan taxa did not differ significantly between biochar-added treatments and CK (Table 2). However, the relative abundance of rare protistan taxa was generally higher in B1.5 and B2.0 than in CK, indicating that biochar application, specifically in B1.5 and B2.0 levels, may have promoted the growth or persistence of rare protistan taxa in the soil. Mantel’s test was also used to investigate associations between soil characteristics and abundant, rare, and intermediate protist species for all treatments. Abundant and intermediate protistan taxa did not correlate with soil attributes, whereas rare protist taxa correlated significantly with soil TN (Figure 4B).

### 3.4 Wheat yield

**Figure 5:**
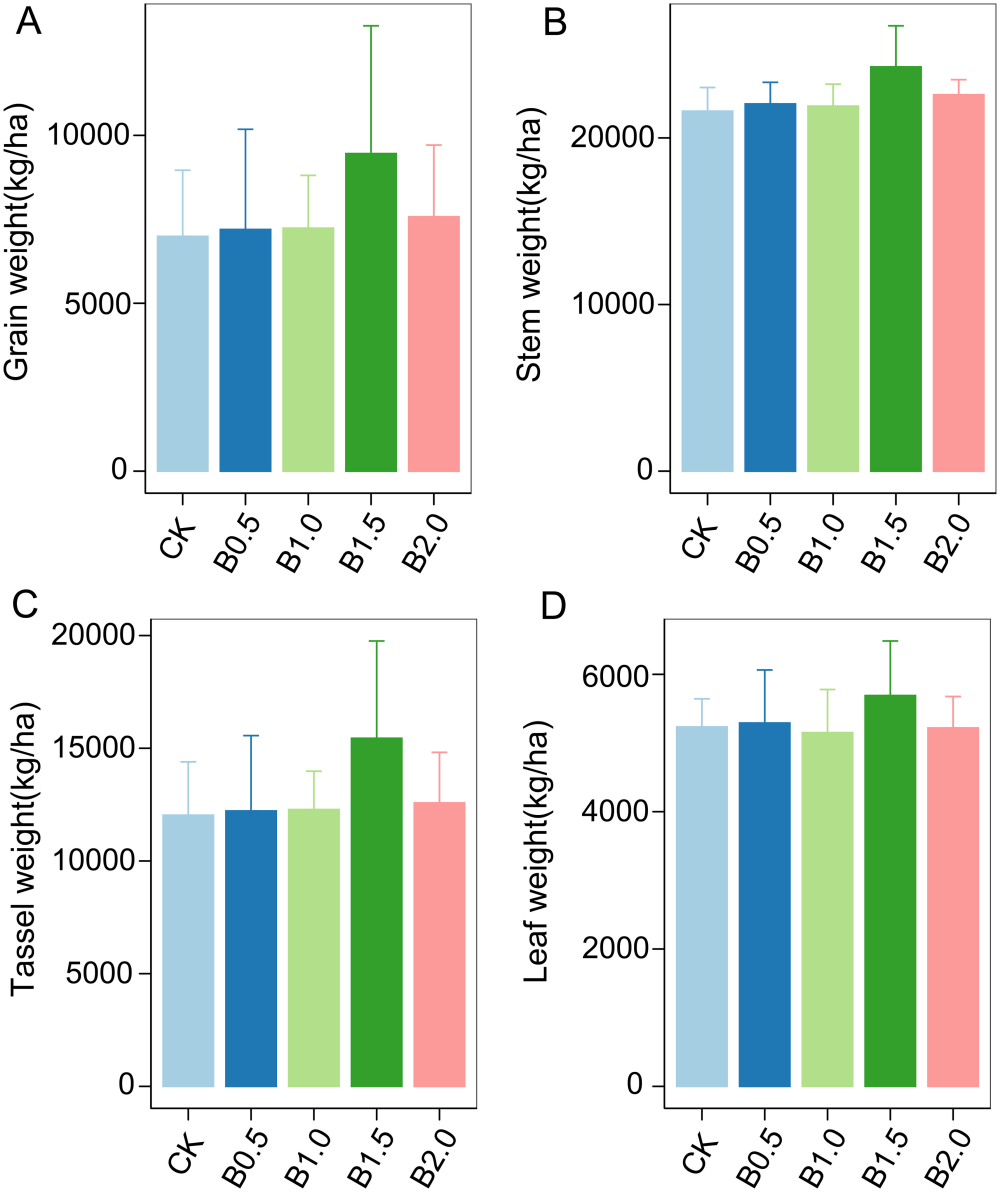
Above-ground biomass during the wheat growth across different treatments are presented. **(A)** is grain weight; **(B)** is stem weight; **(C)** is tassel weight; **(D)** is leaf weight. Error bars indicate that the standard deviation of triplicate analyses. No lowercase letters indicate no significant differences among different treatments (*p* > 0.05). CK: without biochar incorporation, B0.5: Plot amended with 1.14 t ha^−1^ of biochar, B1.0: Plot amended with 2.28 t ha^−1^ of biochar, B1.5: Plot amended with 3.42 t ha^−1^ of biochar, B2.0: Plot amended with 4.56 t ha^−1^ of biochar.

**Figure 6:**
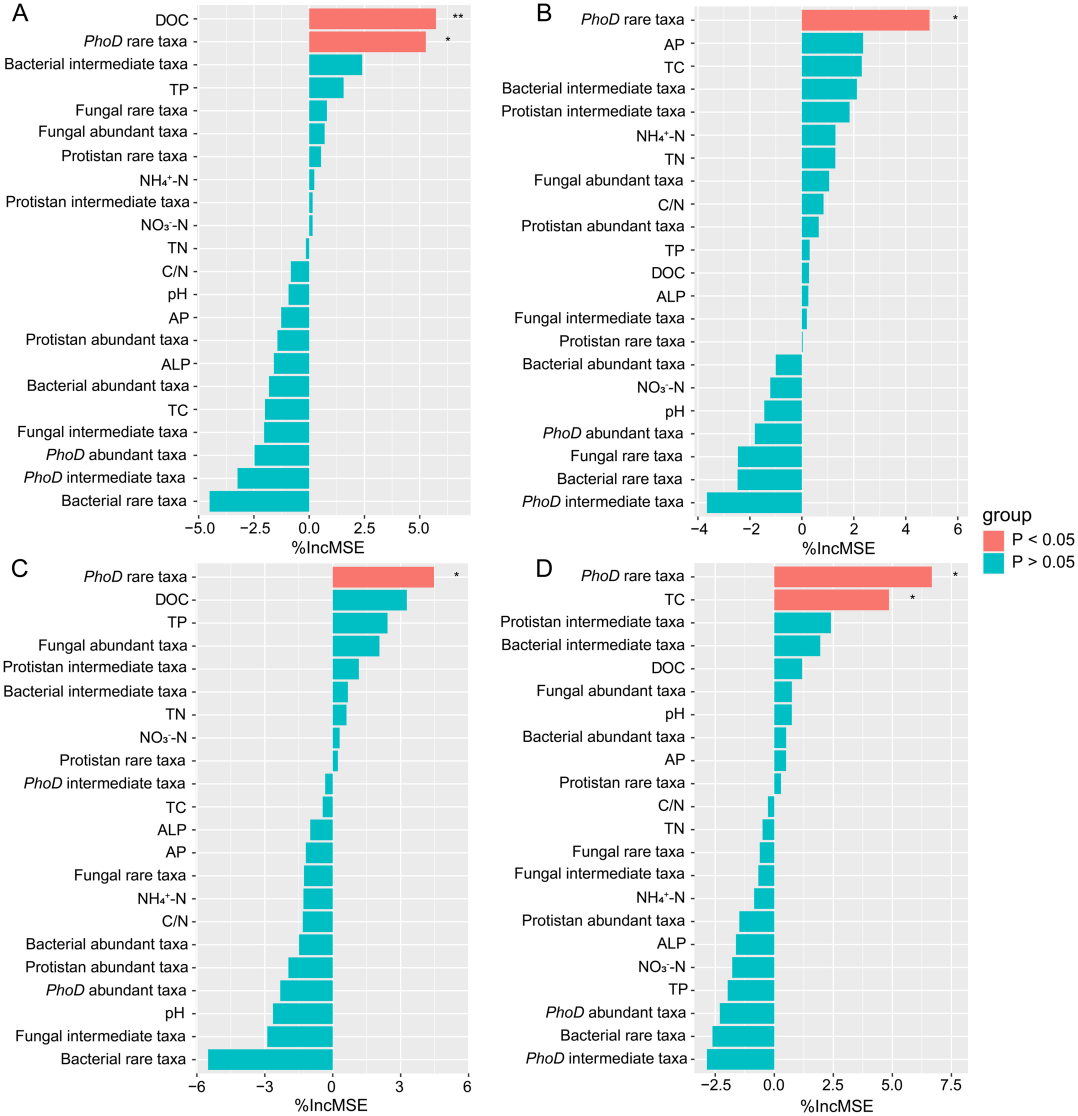
Random forest analysis against abiotic and biotic factors were analyzed for wheat grain weight **(A)**, stem weight **(B)**, tassel weight **(C)** and leaf weight **(D)**. ^**^p < 0.01 and ^*^p < 0.05.

In the third year after biochar application, we measured the grain, stem, tassel, and leaf weights of wheat. Biochar application did not significantly affect wheat growth (Figure 5). However, different biochar treatments slightly increased the grain, stem, tassel, and leaf weights of wheat. The highest increase occurred in B1.5. Although the statistical analysis is not significant, the treatment of B1.5 (3.42 t ha^−1^) has increased the wheat yield by 35% compared to the control without the addition of biochar. We then conducted Manel’s test and random forest regression analyses between soil physicochemical properties, biological factors, and wheat yield. No significant associations occurred between abundance of *phoD* gene copies, the abundance of bacterial 16S RNA gene copies, and their ratios with wheat growth parameters such as grain, stem, leaf, and tassel weights (Supplementary Figure S1). Notably, Mantel’s analysis revealed a significant effect of rare fungal taxon communities on stem weight, whereas rare protistan taxon communities significantly affected grain, tassel, and leaf weights (Figure 4). Random forest regression analyses revealed that rare *phoD*-harboring microbial taxon communities significant influenced grain weight, stem weight, tassel weight and leaf weight of wheat (Figure 6). Furthermore, DOC and TC correlated significantly with grain and leaf weights, respectively (Figure 6).

## 4 Discussion

### 4.1 Effect of biochar on the soil physicochemical properties

Biochar incorporation significantly influenced soil physicochemical properties, although the effects varied depending on the specific property and treatment. Among the five treatments, no significant differences were detected in AP (Table 1). This finding aligns with previous research indicating that the biochar application does not always significantly affect soil AP (An et al., 2022; Yuan et al., 2022). In our study, soil AP content decreased in biochar-added treatments, except for B1.0 (Table 1). The exception in B1.0 might be attributed to the specific interaction between biochar properties and soil conditions at this application rate, which warrants further investigation. For TP, biochar amendment led to a decrease in soil TP in B0.5, B1.5, and B2.0. This reduction could be attributed to biochar’s strong adsorption capacity, as demonstrated by Bornø et al. (2018), which may immobilize phosphorus and reduce its availability in the soil.

Biochar incorporation did not significantly alter soil pH, which contrasts with some previous studies (Tian et al., 2021; Yuan et al., 2022). This discrepancy may be related to the inherently alkaline nature of the original soil (pH 8.78) and the relatively low biochar application rate in this study used. The buffering capacity of the soil likely mitigated any potential pH changes induced by biochar. Although there were no significant differences in soil NH_4_^+^-N content among the five treatments, NH_4_^+^-N levels were lower in biochar-amended treatments compared to the control (CK). This observation aligns with the findings of Lehmann et al. (2011), who reported that biochar’s porous structure and negatively charged functional groups can enhance NH_4_^+^-N adsorption, potentially reducing its availability in the soil solution. This mechanism may explain the lower NH_4_^+^-N content in biochar-amended treatments. Additionally, biochar incorporation altered microbial communities, which might immobilize nitrogen, reducing the available NH_4_^+^-N for crops in the short term.

### 4.2 Effects of biochar on bacterial 16S rRNA and *phoD* genes copies and the ratio of *phoD* to bacterial 16S rRNA gene

In this study, a significant and positive correlation was observed between soil DOC and bacterial 16S rRNA gene copy number (Supplementary Figure S1). This suggests that labile carbon, represented by DOC, directly influences soil bacterial abundance. This finding is consistent with previous studies indicating that biochar can provide a carbon source, particularly labile C, for microorganisms, thereby impacting bacterial abundance (Cross and Sohi, 2011; Rutigliano et al., 2014; Huang et al., 2021). For instance, Dai et al. (2018) reported that labile carbon from biochar affects microbial biomass in farmland soil. Additionally, Luo et al. (2017b) utilized isotopic tracing methods to demonstrate that the labile carbon fraction of biochar strongly correlates with microbial respiration, which in turn influences bacterial abundance. These findings support our results and highlight the importance of labile carbon in regulating microbial dynamics.

Furthermore, soil TN correlated positively with the ratio of *phoD* gene abundance to bacterial 16S rRNA gene abundance (Supplementary Figure S1). This relationship may be explained by the fact that N amendments can increase P fertilizer availability, and N incorporation prompts microorganisms to invest in phosphatase production (Arenberg and Arai, 2019). A meta-analysis by Margalef et al. (2017) also revealed that TN is a key factor influencing regional variations in phosphatase activity. Additionally, ammonium has been shown to significantly promote alkaline phosphatase activity generated by *phoD*-containing microorganisms (Arenberg and Arai, 2019). Dai et al. (2018) reported that biochar influences soil P cycling by affecting the activity and communities of P-solubilizing microbes. Microbial biomass size, activity, and reproduction are determined based on soil nutrient availability (Fu et al., 2021; Xue et al., 2023). Moreover, Zhang et al. (2023) reported that *phoD* abundance correlates positively with TN, a notable environmental parameter affecting *phoD*-harboring bacteria. These findings suggest that biochar-induced changes in soil N availability may indirectly influence P cycling by modulating the activity and abundance of *phoD*-harboring microbes.

### 4.3 Effects of biochar on abundant, intermediate, and rare bacterial, fungal, protistan, and *phoD*-harboring microbe taxa after biochar incorporation

The Adonis results indicated that biochar incorporation had no significant impact on the composition of abundant, intermediate, and rare bacterial taxa or abundant and intermediate fungal taxa. This finding contrasts with some previous studies, which have reported varying effects of biochar on soil microbial communities depending on application rates, biochar type, and soil conditions (Lehmann et al., 2011; Gul and Whalen, 2016; Dai et al., 2018; Dai et al., 2021; Huang et al., 2021). In our study, the relatively low biochar application rates (0–4.56 t ha^−1^) may explain the lack of significant effects on these microbial groups (Zhang et al., 2021). Notably, rare fungal taxon exhibited significant differences among treatments, suggesting that they are more responsive to biochar addition, which often occupy narrow ecological niches and are more susceptible to environmental changes.

For *phoD*-harboring microbes, Adonis analysis revealed that biochar addition significantly altered the structure of intermediate and rare taxa. The finding aligns with Wei et al. (2019), who implicated rare taxa in mediating soil phosphorus mineralization. Mantel’s analysis further revealed a significant correlation between AP and intermediate *phoD*-harboring taxa (Supplementary Figure S2B). This suggests that intermediate *phoD*-harboring taxa may play a key role in P cycling under biochar-amended conditions. Additionally, rare protistan taxa were significantly affected by biochar addition, with their community structure showing distinct changes in responses to treatment (Figure 3L). Rare protists, despite their low abundance, are increasingly recognized as important components of soil ecosystems (Jousset et al., 2017). They play a crucial role in maintaining the ecological balance and resilience of soil ecosystems and contribute to nutrient cycling (Jousset et al., 2017). Phagotrophic protists among them are key factors in controlling bacterial and fungal communities in soil (Xiong et al., 2018). Through predation, they release nutrients immobilized in microbial biomass, which could accelerate nutrient turnover and increases plant nutrient uptake (Xiong et al., 2018). Protists also contribute significantly to nutrient cycling, being involved in organic matter degradation and carbon fixation processes, with their activities facilitating nutrient transformation in the soil (Xiong et al., 2020). The significant correlation between rare protistan taxa and soil TN (Figure 4B) further highlights their potential role in N dynamics.

### 4.4 Effect of biochar on the wheat yield

Biochar application did not significantly increase wheat yield compared to in the control (CK) (Figure 5). This study is consistent with some previous studies, which have reported limited or no yield response to biochar application (Ye et al., 2019; Xu et al., 2015; Bai et al., 2022). The lack of a significant yield increase may be attributed to the low nutrient content of the maize straw biochar used in this study, which may have limited its ability to enhance soil fertility and plant growth. Additionally, the relatively low application rates of biochar (up to 4.56 t ha^−1^) may have been insufficient to induce significant changes in soil properties or crop performance.

Although the statistical analysis is not significant, the treatment of B1.5 (3.42 t ha^−1^) has increased the wheat yield by 35% compared to the control without the addition of biochar. Random forest regression analysis indicated that DOC and TC significantly affected crop growth (Figure 6). However, DOC and TC concentrations did not differ significantly among treatments, suggesting that other factors, such as microbial community composition, may have played a more important role in influencing yield. Notably, rare fungal and protistan taxa were significantly correlated with wheat growth parameters, including stem weight, grain weight, tassel weight, and leaf weight (Figure 4). These findings highlight the potential importance of rare microbial taxa in regulating crop performance and suggest that biochar-induced changes in microbial communities may indirectly influence yield.

## 5 Conclusion

Our findings indicate that biochar addition to fluvo-aquic soil altered soil physicochemical properties, particularly TP and NH_4_^+^-N. The application of biochar at 3.42 t ha^−1^ slightly increased wheat yield, although the effect was not statistically significant. Rare *phoD*-harboring taxa and DOC were identified as key factors influencing wheat grain yield. Additionally, rare protistan taxa significantly correlated with wheat growth, highlighting their potential role in nutrient cycling and crop performance. Future studies should further investigate the functional roles of rare *phoD*-harboring and protistan taxa to fully understand their interactions with soil P cycling and crop growth in biochar-amended soil. Moreover, longer-term field trials are needed to validate these findings and explore the potential benefits of higher biochar application rates.

## Data Availability

The datasets presented in this study can be found in online repositories. The names of the repository/repositories and accession number(s) can be found in the article/Supplementary material.

## Footnotes

^1^ https://www.r-project.org
