# Supplementary material for: Biochar amendment alters rare microbial taxa and enhances wheat growth in alkaline farmland: insights into soil microbiome dynamics
Source: Front Microbiol. 2025 May 21;16:1563712. doi: 10.3389/fmicb.2025.1563712 (PMC12136494; doi:10.3389/fmicb.2025.1563712)
Supplement: Supplementary file 1 [file Data_Sheet_1.pdf]

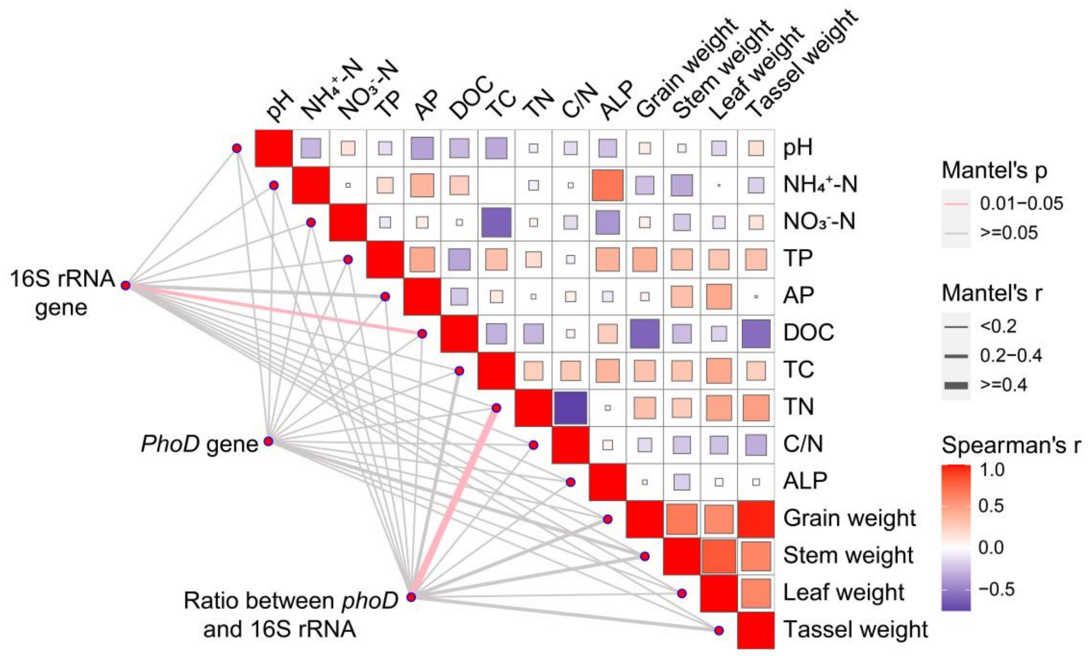

Figure S1 Pairwise comparisons of soil attributes are shown in the upper right corner, with a color gradient denoting Spearman's correlation coefficient. 16S rRNA gene copy numbers, *phoD* copy numbers and the ratio of *phoD* to 16S rRNA gene copy numbers are related to each soil attribute and wheat yield by Mantel test. Edge color denotes the statistical significance and edge width corresponds to the Mantel's r statistic for the corresponding distance correlations. TP, total phosphorus; AP, available phosphorus; TN, total nitrogen; TC, total carbon; NO_3^--N, dissolved nitrate nitrogen; NH_4^+-N, dissolved ammonium nitrogen; DOC, dissolved organic carbon; ALP, potential alkaline phosphatase activity.

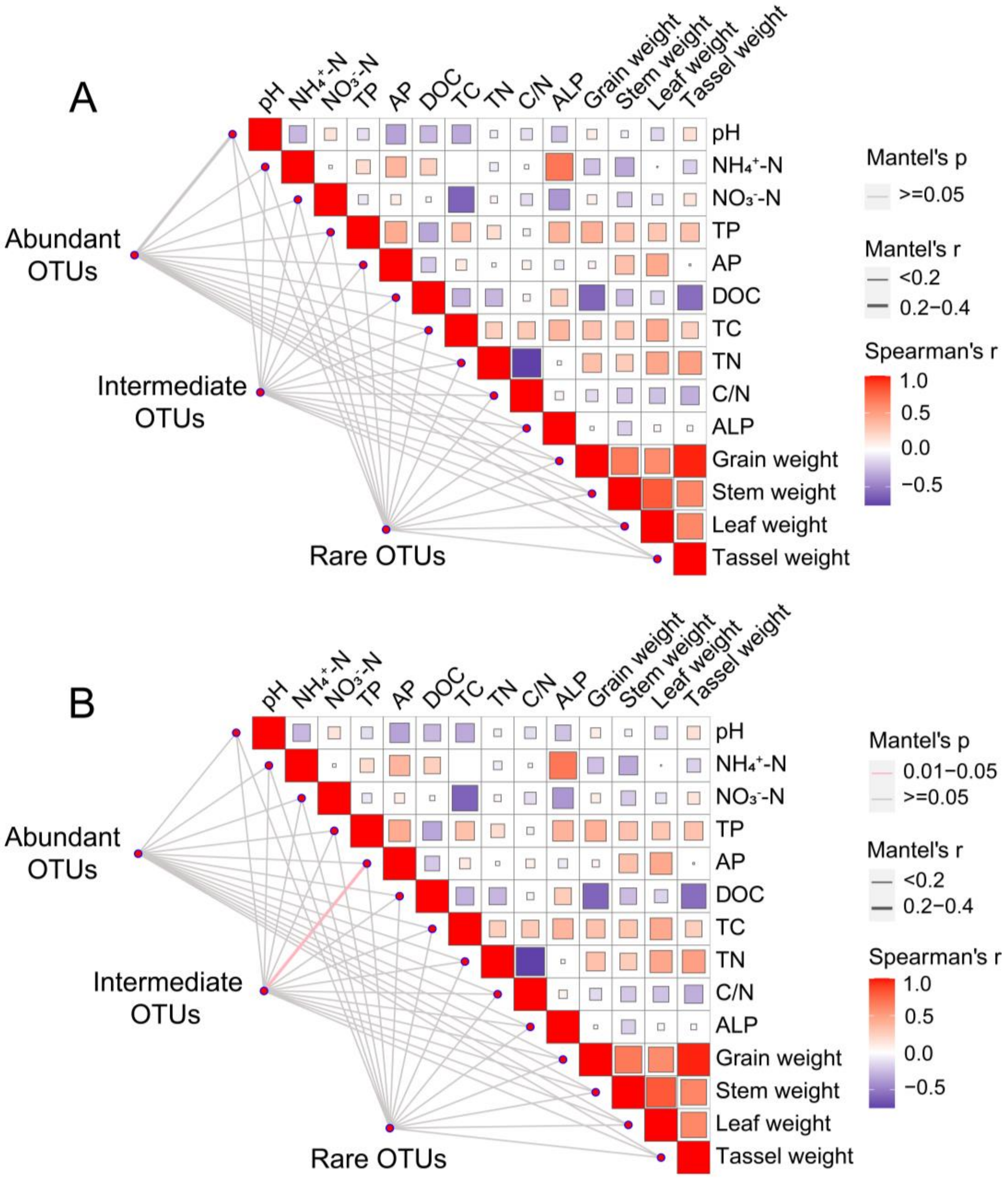

Figure S2 Pairwise comparisons of environmental factors are shown in the upper right corner, with a color gradient denoting Spearman's correlation coefficient. Abundant (relative abundance>0.1%), intermediate (0.01%<relative abundance<0.1%) and rare OTUs (relative abundance<0.01%) of bacterial (A) and *phoD*-harboring microorganisms (B) are related to each soil attribute and wheat yield by Mantel test. Edge color denotes the statistical significance and edge width corresponds to the Mantel's r statistic for the corresponding distance correlations. TP, total phosphorus; AP, available phosphorus; TN, total nitrogen; TC, total carbon; NO_3^--N, dissolved nitrate nitrogen; NH_4^+-N, dissolved ammonium nitrogen; DOC, dissolved organic carbon; ALP, potential alkaline phosphatase activity.

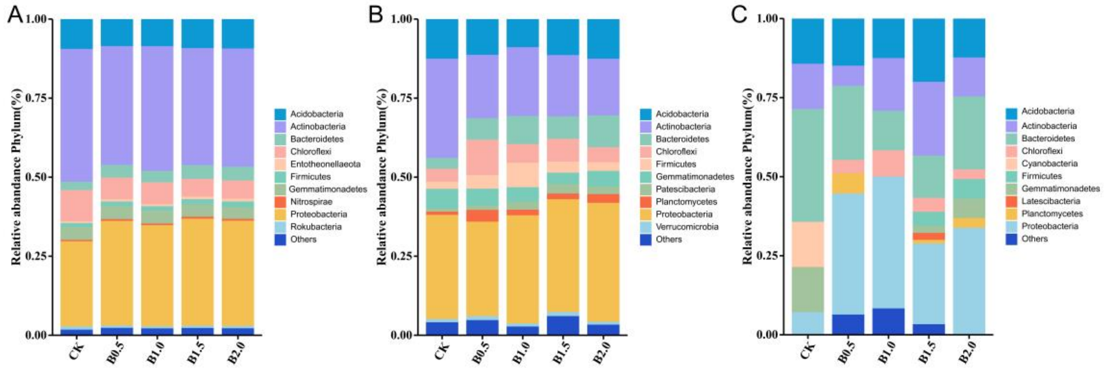

Figure S3 Distribution of bacterial abundant taxon community composition (A), distribution of bacterial intermediate taxon community composition (B) and distribution of bacterial rare taxon community composition (C).

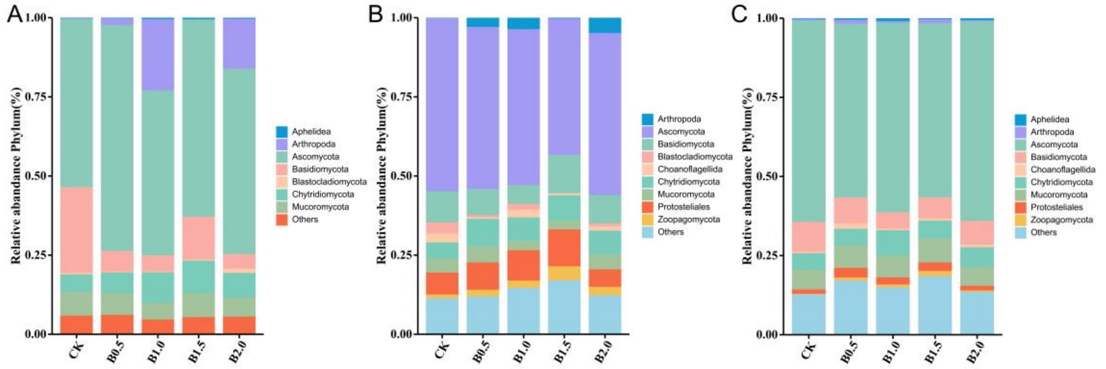

Figure S4 Distribution of fungal abundant taxon community composition (A), distribution of fungal intermediate taxon community composition (B) and distribution of fungal rare taxon community composition (C).

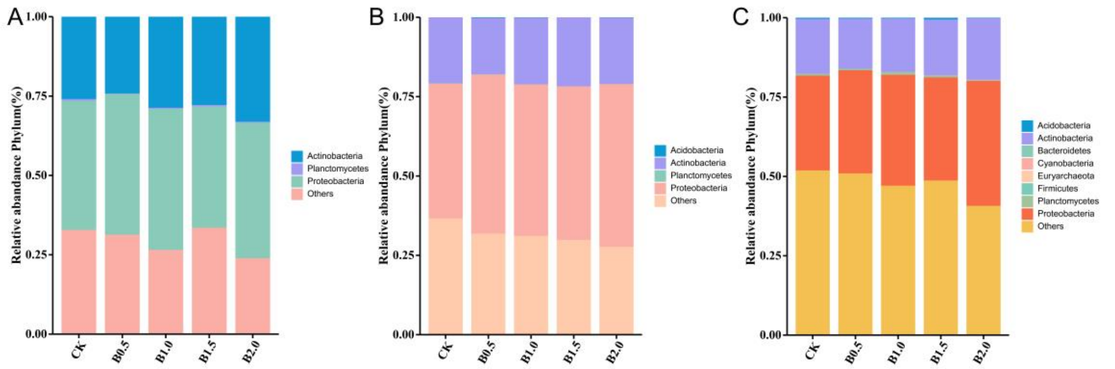

Figure S5 Distribution of *phoD*-harboring abundant taxon community composition (A), distribution of *phoD*-harboring intermediate taxon community composition (B) and distribution of *phoD*-harboring rare taxon community composition (C).

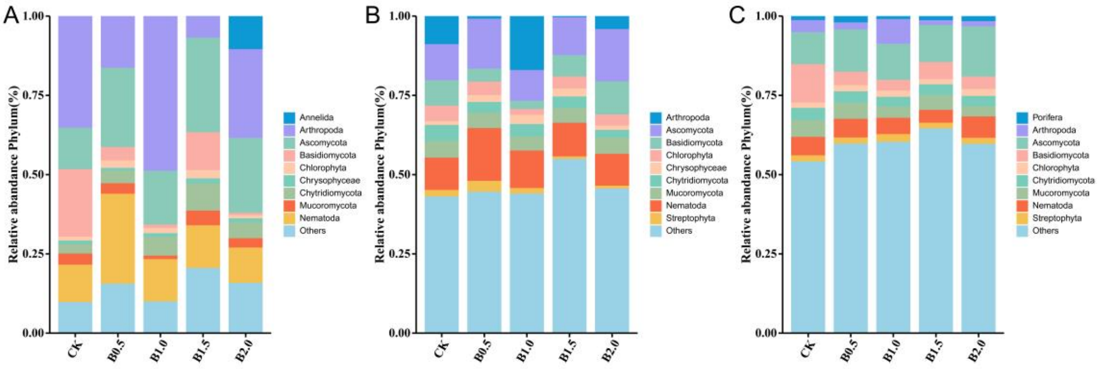

Figure S6 Distribution of protistan abundant taxon community composition (A), distribution of protistan intermediate taxon community composition (B) and distribution of protistan rare taxon community composition (C).

Table S1 Details of primer sets and thermal conditions used in the qPCR

Target genes	Primers	Sequence (5'-3')	Length of fragments	Thermal profile	References
phoD	F733<br>R1083	TGGGAYGATCAYGARGT<br>CTGSGCSAKSACRTTCCA	350	95°C for 3min;95°C for 30s, 55°C for 30s (35cyles)	(Chen *et al.*, 2019)
Bacterial 16S rRNA genes	1369F<br>1492R<br>Probe TM 1389F	GGGGTGCGGTCYTTNARYTC<br>ACGGCTACCTTGTTACGACTT<br>CTTGTACACACCGCCCGTC	123	95°C for 3min;95°C for 15s, 56°C for 30s (35cyles)	(Jia *et al.*, 2014)

Table S2 Details of primer sets and thermal conditions used in the high-throughput sequencing

Target genes	Primers	Sequence (5'-3')	Length of fragments	Thermal profile	References
phoD	F733<br>R1083	TGGGAYGATCAYGARGT<br>CTGSGCSAKSACRTTCCA	350	95°C for 3min;95°C for 30s, 55°C for 30s, 72°Cfor30s (35cyles);<br>72°C for 10 min	(Chen *et al.*, 2019)
Protist	FW-TAReuk454FWD1<br>REV-TAReukREV3	CCAGCA(G/C)C(C/T)GCGGTAATTCC<br>ACTTTCGTTCTTGAT(C/T)(A/G)A	418	95°C for 5min;94°C for 30s, 57°C for 45s, 72°C for 60s (10cyles)<br>94min for 30s, 45°C for 45s, 72°C for 60s (25cyles);<br>72°C for 2 min	(Stoeck *et al.*, 2010)
Fungi	FW-F817<br>REV-R1196	TTAGCATGGAATAAT(A/G)(A/G)AATAGGA<br>TCTGGACCTGGTGAGTTTCC	379	95°C for 3min;95°C for 30s, 55°C for 30s, 72°Cfor45s (35cyles);<br>72°C for 10 min	(Rousk *et al.*, 2010)
Bacterial 16S rRNA genes	338F<br>806R	ACTCCTACFGGGAGGCAGCA<br>GGACTACHVGGGTWTCTAAT	468	95°C for 5min;95°C for 30s, 55°C for 30s, 72°Cfor30s (35cyles);<br>72°C for 5 min	(Fadeev *et al.*, 2021)

Table S3 Ratio and Relative abundance of abundant, intermediate and rare taxa of soil bacteria, fungi, protist and *phoD*-harboring microbes

	Abundant taxa		Intermediate taxa		Rare taxa	
Taxa	Ratio	Relative Abundance	Ratio	Relative Abundance	Ratio	Relative Abundance
Bacteria	0.43%	21.82%	6.20%	47.75%	93.37%	30.43%
Fungi	2.41%	89.67%	7.95%	6.70%	89.64%	3.63%
Protist	1.60%	65.74%	10.74%	21.84%	87.67%	12.42%
phoD-harboring microbes	1.65%	56.25%	10.17%	28.33%	88.17%	15.42%

Ratio: the OTUs which belong to abundant taxa, intermediate taxa or rare taxa to all OTUs.

Relative abundance: the relative abundance of abundant, intermediate or rare taxa in all OTUs of all samples.

References

Chen, X., Jiang, N., Condron, L.M., Dunfield, K.E., Chen, Z., Wang, J., Chen, L., 2019. Soil alkaline phosphatase activity and bacterial *phoD* gene abundance and diversity under long-term nitrogen and manure inputs. Geoderma 349, 36-44. https://doi.org/10.1016/j.geoderma.2019.04.039.

Fadeev, E., Cardozo-Mino, M.G., Rapp, J.Z., Bienhold, C., Salter, I., Salman-Carvalho, V., Molari, M., Tegetmeyer, H.E., Buttigieg, P.L., Boetius, A., 2021. Comparison of Two 16S rRNA Primers (V3-V4 and V4-V5) for Studies of Arctic Microbial Communities. Front. Microbiol. 12, 637526. https://doi.org/10.3389/fmicb.2021.637526.

Jia, Y., Huang, H., Chen, Z., Zhu, Y.G., 2014. Arsenic uptake by rice is influenced by microbe-mediated arsenic redox changes in the rhizosphere. Environ. Sci. Technol. 48, 1001-1007. https://dx.doi.org/10.1021/es403877s.

Rousk, J., Baath, E., Brookes, P.C., Lauber, C.L., Lozupone, C., Caporaso, J.G., Knight, R., Fierer, N., 2010. Soil bacterial and fungal communities across a pH gradient in an arable soil. ISME. J. 4, 1340-1351. https://doi.org/10.1038/ismej.2010.58.

Stoeck, T., Bass, D., Nebel, M., Christen, R., Jones, M.D., Breiner, H.W., Richards, T.A., 2010. Multiple marker parallel tag environmental DNA sequencing reveals a highly complex eukaryotic community in marine anoxic water. Mol. Ecol. 19 Suppl 1, 21-31. https://doi.org/10.1111/j.1365-294X.2009.04480.x.
